# Patient-stimulated fall prevention screening in primary care: analysis of provider coding changes

**DOI:** 10.1186/s12875-023-02154-x

**Published:** 2023-09-14

**Authors:** Ryan Moran

**Affiliations:** San Diego Department of Medicine, Department of Family Medicine, University of California, 8899 University Center Lane, St 4000, La Jolla, CA 92037 USA

**Keywords:** Falls, Screening, Primary care, Health services research, Aging

## Abstract

**Background:**

The Centers for Disease Control and Prevention (CDC) has developed an evidenced based clinical screening tool, Stopping Elderly Accidents, Deaths & Injuries (STEADI) but penetration into routine clinical practice has been slow. To increase screening for falls and fall risk in an internal medicine primary care practice, a patient-centered screening program was integrated into a busy academic clinic.

**Methods:**

Over a three month period, Patients were invited to self-screen via a large poster in the waiting room, and complete a STEADI Staying Independent questionnaire, and discuss findings with their healthcare provider. Fall Prevention Booklets were made readily available in clinic exam rooms. Questionnaires and fall prevention booklets, were uniquely numbered, and Epic Slicer-Dicer reports were utilized to evaluate falls screening-related ICD-10 codes determined a priori. Generalized linear modeling calculated difference-in-difference compared with other clinics without this program for rates of coding for fall-related diagnosis codes.

**Results:**

In three months, 255 questionnaires were taken; only 5 (2%) were returned for later review. 110 booklets were disseminated from clinic exam rooms. The absolute difference-in-difference in ICD-10 coding was 0.7% compared to other clinics in the same practice, and year before. Generalized linear modeling showed a 4.7% increased impact in screening-related ICD-10 codes, which was statistically significant (*P* =  < .0001) without reported disruption to clinical workflows.

**Conclusion:**

There are indicators that patient-centered selective screening at a busy academic practice may have resulted in an increase in falls-related ICD-10 coding. Clinical integration of this program was well received.

## Background

In 2016, US residents over age 65 totaled almost 50 million, an increase of over 40% since just 2000 [1]. Between one-quarter and one-third of community-residing adults aged 65 or older, and one-half of those over 80 fall annually [2, 3]. Falls are a major cause of morbidity and mortality, as unintentional falls remains the most common cause of nonfatal injury in the United States, and proportionally increases as individuals age [4]. Of concern, fall-related mortality has increased over 30% just between 2007 to 2016 [1].

Fall risk is multifactorial, and often the result of interaction between intrinsic and extrinsic factors. Intrinsic factors include vision problems, cognitive/neurological impairment, depression, side effects from medications, and/or multiple medication use, hypotensive episodes, muscular weakness, loss of flexibility, and deficits in balance, mobility and gait [5–9]. Extrinsic factors include home hazards, uneven terrain, poor lighting in the home and neighborhood, inappropriate footwear, and distractions such as those occurring on a busy street. Further, a single fall predicts recurrent falls: between 10 and 44% of elderly patients with a history of falls will sustain additional falls [5, 10–13].

Many falls are preventable, and the CDC has developed an evidenced-based, multifactorial clinical approach to identify those at risk for falls, to help assess immediate/known concerning flags, and to intervene, often including referrals for community-based fall-prevention programs [14, 15]. The Stopping Elderly Accidents, Deaths and Injuries (STEADI), toolkit has been shown to be a feasible approach as part of routine clinical care [16]; however penetration into practice has been low. Major barriers reported by physicians to implementing STEADI are time constraints, poor reimbursement for falls screening, and that toolkit utilization does not easily fit into a Medicare wellness visit [16, 17]. Because of this, only around one-third of older adults report being asked about falls or fall-risk, and similarly only around a third of those who fall report discussing this with their healthcare provider [18, 19]. Thus, there remains significant quality improvement opportunity to better understand how to meaningfully integrate fall-screening and prevention strategies within routine primary care practice.

In this context, the overall goal of this project was to test the feasibility of incorporating a modified STEADI screening algorithm into busy academic practices by positioning patients to be better equipped to discuss falls-prevention with their providers, thereby involving them as a champion for fall-screening and risk reduction.

## Methods

A quality improvement/evidence-based practice intervention was initiated to improve fall-risk screening and discussion of falls-prevention strategies between June and August 2019 within the University of California, San Diego Health System. This intervention was performed at the largest and busiest of the system’s four academic internal medicine practices, which has a patient volume of between 120–200 patients daily and has around 10 attending physicians on any given week-day, with many trainees including medical students and resident physicians. This program aimed to target older adults to self-select for fall-risk with a modified STEADI screening tool for fall risk, and evaluate the impact on medical coding at the clinic visit via ICD-10 code evaluation (Table 1). As there are numerous and redundant ICD-10 codes related to fall-concerns, even utilizing mechanisms to group appropriately (e.g. SNOMED), these ICD-10 codes were selected despite being nonexclusive as they were previously utilized in another study looking at integrating STEADI within primary care [16]. While this previous study utilized an Epic-based Smartset, a supported Epic build to streamline provider selection of diagnosis codes and referrals/orders (whereby clinicians were subjected to predetermined clickable associations for diagnoses, making understanding changes in coding patterns much easier), Smartsets were used infrequently in the clinical practice. Therefore, these 10 codes were selected to serve as an approach to estimate and evaluate for coding changes through this project of clinicians without directly influencing or altering clinical workflows. Specifically, there was no education or instruction to any providers to change any documentation or clinical practice, and providers were unaware that these codes being used throughout this project.

**Table 1:** ICD Coding for fall risk

	**ICD10 Code**	**Diagnosis**
1	Z91.81	History of falling
2	R26.2	Difficulty walking, not elsewhere classified
3	I95.1	Orthostatic hypotension
4	R29.6	Repeated falls
5	H53.9	Unspecified visual disturbance
6	R26.9	Unspecified abnormalities of gait and mobility
7	R26.89	Other abnormalities of gait and mobility
8	I95.9	Hypotension, unspecified
9	R27.*/R27.9	Unspecified lack of coordination
10	R29.3	Abnormal posture
11	M62.81	Muscle weakness, generalized

**Fig. 1:**
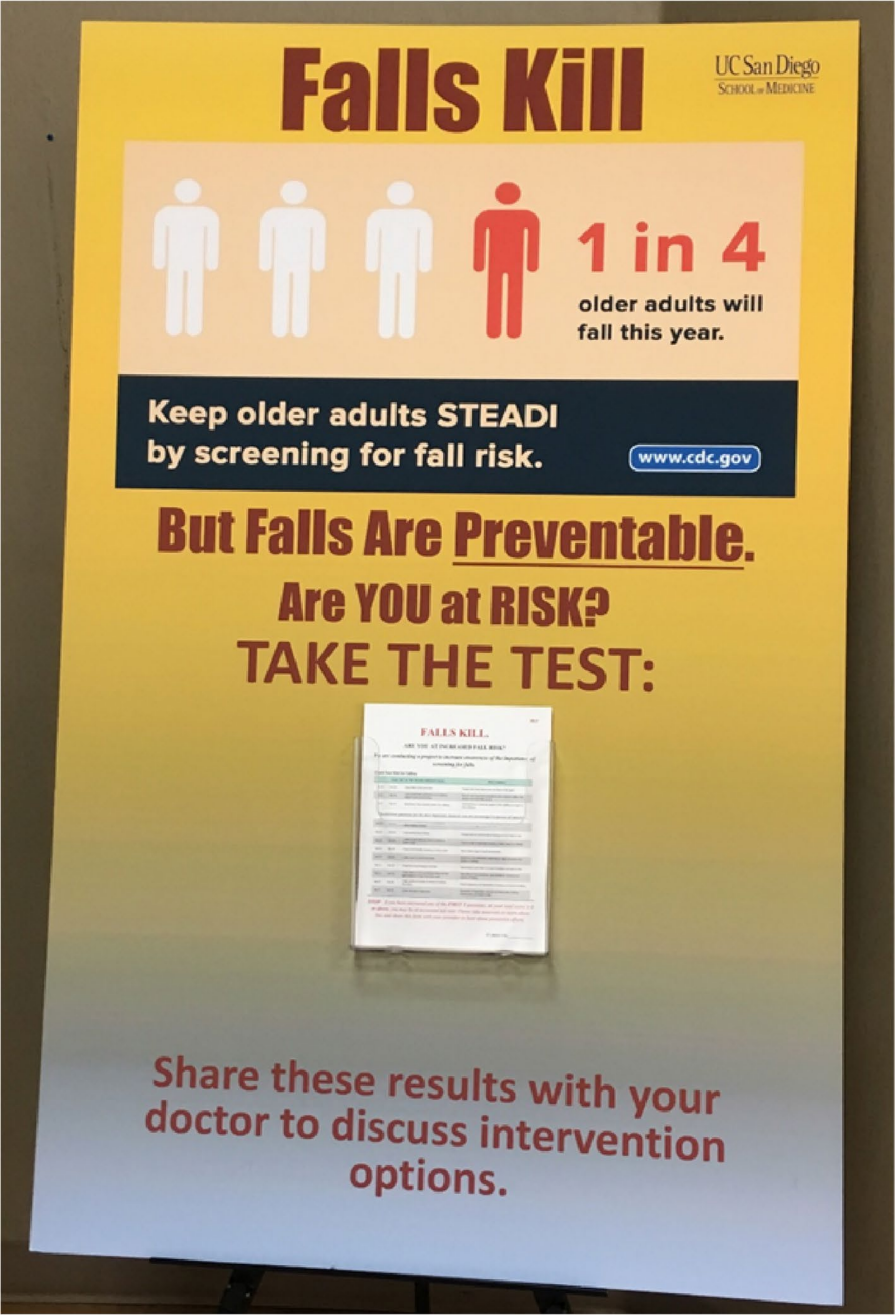
Waiting Room Figure inviting patients to fill out STEADI questionnaire

To highlight the risk for falls, an approximately 3’ × 5’ poster board was generated using available resources from the CDC—modified with language to heighten awareness (e.g.: “Falls Kill”)—and was placed at the clinic doorway showcasing fall-related concerns. On this poster, STEADI *Staying Independent* questionnaires were numbered, and made available on the poster board, for patients to take and complete. Marking a ‘yes’ to any of three questions (‘I have fallen in the past year’; ‘I am worried about falling’; and ‘Sometimes I feel unsteady when I am walking’), or a score of 4 or more based on positive responses would traditionally been deemed a ‘positive screen’ and would result in further evaluation and screening physical function, vision, and orthostatic vitals to better qualify risk of falls [20]. However, as this pilot aimed to increase clinical integration of screening, and in an effort to minimize clinic workflow disruption, individuals who self-selected the questionnaire and filled it out were only requested to share the results with their healthcare provider. In addition, the first three questions were clearly prioritized, as they have been shown to identify 95% of high risk individuals [21] by themselves. The brochure highlighted sharing findings with their healthcare providers if the findings indicated a score of 4 or more (Fig. 1).

Booklets containing evidence-based CDC fall prevention strategies including orthostasis/position changes suggested for dizziness, brief and general information on medications, and chair-based exercises, as well as community resources including free and low-cost fall prevention exercise programs were created, and made available in all exam rooms throughout the clinic. Materials selected were under guidance from the clinician involved in this project with exercise science and health promotion experience, as well as with input from exercise scientists and public health experts. These booklets served two purposes: (1) to allow a more thorough discussion regarding fall-prevention strategies including having materials available for patients to take home; and (2) to allow for providers to provide resources in the event there is insufficient time in a clinic encounter to fully explore fall-risk and prevention strategies. Because of limitations in resources from the local health department, resources were only available in English for this project. All exam rooms in the clinic were evaluated every two weeks to assure a continuous supply of five booklets, which allowed for the numbers missing to approximate clinical information dissemination.

All questionnaires returned to the clinic staff were collected. Medical record coding review was conducted via Epic Slicer-Dicer, to evaluate certain fall-related ICD-10 codes (Table 1).

Reports generated included the total number of patients seen by clinic site (at the 4 internal medicine academic practices) by month aged 65 and older seen at the clinic site, as well as the total number of patients seen by the clinic site aged 65 or older with a corresponding ICD-10 ‘screen’ code. The percent of patients screened was calculated as those having at least one of the ICD-10 codes entered at their clinic appointment, over the total number of patients 65 years and older. This percentage was recorded in Excel to identify unique patient-experience encounters, by clinic site, and by time. Slicer-dicer reports were generated for the previous year for all coding at these clinics, but analysis was limited to the three months directly before intervention, the three months of intervention, and the same three-month period 1 year prior to the intervention to control for seasonality changes in coding and falls.

Difference-in-difference modeling was utilized to determine impact of the study intervention. Coding for modeling was determined by if patients were screened or not (1 for screen,0 if no corresponding ICD-10 code at visit), clinic site (1 for intervention, 0 for other clinic site), and time (1 time of intervention, 0 the same time frame the year before). Generalized linear modeling (SAS 9.4 GENMOD procedure) was utilized to determine significance with change estimates from the intervention determined by the least square means.

This project was determined and approved to be appropriate as an Evidence-Based Practice/Quality Improvement Project, by the UCSD IRB and therefore IRB exempt for full review.

## Results

Between June and August, 2019, 255 questionnaires were taken; however only 5 (2%) were returned for review limiting chart review opportunity and characterizing screened individuals. Within the clinic, 110 booklets for falls prevention information were taken by patients from exam rooms. Including the same three months from the year before (6 months total), there were a total of 43,285 clinical encounters at all 4 academic clinical sites for individuals over 65. Approximately 54% of clinical encounters occurred at the clinic site of the intervention (N = 23,549) in these total of 6 months, and about 60% (14,071) of those were in the subjected time frame of this project (3 months). Just under 2% of those encounters screened with a STEADI form (1.8%).

**Fig. 2:**
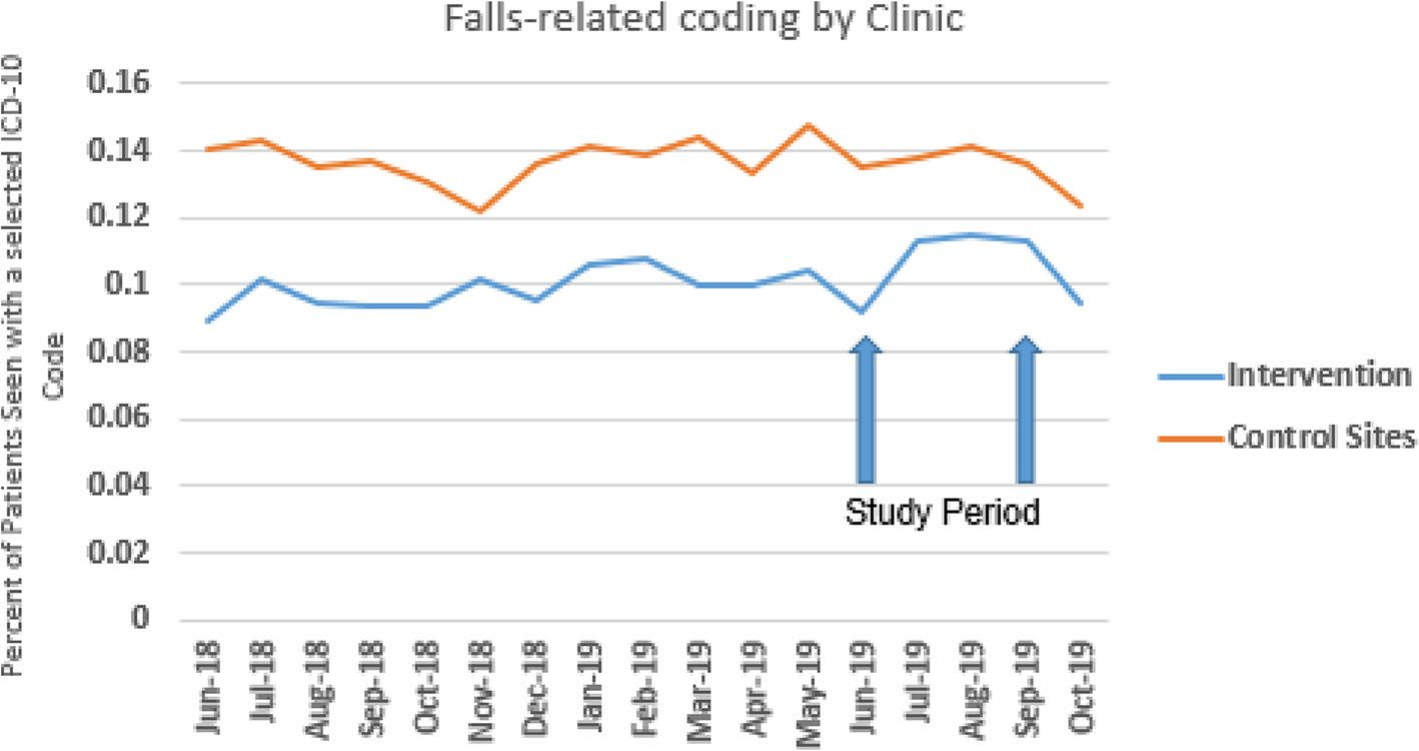
Time series indicating screening rates (ICD10 codes) all four clinical sites (Intervention vs 3 other sites combined) at the UCSD Academic clinical primary care practice. Denominator = % of patients seen at respective clinical site aged 65–110, Numerator = % of those with an ICD-10 code reflective of falls-screening or intervention

Across the system, there was an average ICD-10 coding rate of 11% in patients from the selected fall-related codes. Comparing the clinic with the falls screening program to other clinics within the integrated system, and comparing against the year before, the absolute differential in coding was 0.74% (clinic of interest difference of 0.6%, and at other clinics -0.14%). Modeling difference-in-differences showed a 4.7% increase in screening-related ICD-10 codes, which was statistically significant (*P* =  < 0.0001) (Fig. 2). This translates to about 75 additional entries of the selected ICD-10 codes than what would be expected within this period.

There was no reported disruption to clinical workflows, and no concerns reported from clinic staff, physicians, or patients. Informal evaluation from both staff and physicians reported the material availability was extremely useful to begin conversations about falls, or provide tangible action plans to decrease fall and fall risk.

## Discussion

In this 3-month pilot, patient-centered selective screening at a busy academic practice resulted in an increase in ICD-10 coding by healthcare providers related to falls and fall-risk. While small, this change in coding is meaningful, as it implies behavioral change by both patients and providers. Notably, the baseline use of the selected codes was considerably less than in other clinics in the comparison, as noted in Fig. 2. Although the reasons are likely multifactorial (for example, one of the other clinics included a Geriatric medicine practice), the persistence of the difference across time enhances interpretation of coding changes as shown in this pilot. This intervention differed from previous STEADI-based falls prevention programs [21, 22] in that the aim was to change patient behavior rather than clinic or provider operations; while provider-intiated discussion as a result of this pilot was possible, given the presence of poster (Fig. 1) in the lobby, this is thought to be unlikely given no clinicians or providers were made aware of the ongoing pilot or use of the specific ICD-10 codes. While almost no screening questionnaires were returned, limiting chart review opportunity, this is likely as clinic staff (medical assistants) were requested to do this without knowledge or context of this ongoing project, and many were likely removed from the room by patients or shredded with other patient-rooming materials by clinic staff.

Strengths of this trial are the practical implementation approach, which can be easily and readily expanded or adopted at other clinical sites. Although relatively few falls-booklets were disseminated in the context of the total patient volume through our clinics, this still was a substantial improvement to clinic processes before this program. The design of this program was to empower the patient as the driver of fall-prevention discussion, not the medical provider. This is important, as clinical practice is busy, and while numerous primary-care oriented screenings occur and are important, their impact likely lose value without patients being fully invested. Finally, this intervention did not change or alter routine clinical practice or processes, and provided value-added service to both patients and providers, many of whom informally commented how useful the booklets were for their patients.

Limitations of this trial include the lack of returned questionnaires, which severely affected the ability to perform chart review to gain valuable information regarding individuals who opted to fill out the STEADI screening form, and to be able to follow up on subsequent clinical encounters regarding falls and fall risk. In addition, ICD-10 coding is only a surrogate marker for clinical behavior, as the context is often lost oversimplifying discussion and interaction between a patient and healthcare provider. The use of only 11 ICD-10 codes (Table 1) was incomplete and less comprehensive in capturing the effect of our intervention. For example, many ICD-10 codes (such as R42, *dizziness and giddiness* among many others) were not included, and in busy clinical encounters coding selection may be hurried or not fully capture a clinician’s intention. As mentioned, the study by Casey et al. [16] utilized Epic ‘Smartsets’ which were not routinely used in the clinical practice of this project, so relying on individual provider choice documentation likely only captured a part of those available in the context of a fall-risk discussion. The decision to not use a ‘Smartset’ came at the trade-off of decreased clinical mandates/workflow disruptions. Finally, by not completing the entire STEADI algorithm, which would identify higher risk individuals, this project may have inappropriately provided information on community resources instead of one-on-one treatments (such as physical therapy, which may have been more appropriate). While this possibility exists, it should be noted all free and low cost community resources in the booklet disseminated are overseen by trained professionals, and therefore likely pose little to no risk to patients interested who may not be appropriate as they likely would be turned away. In addition, given no change in clinician workflow was suggested, nor were clinicians aware of the coding being selected, it is unlikely for these higher risk individuals would not get additional appropriate counseling and recommendations.

A focus on patient-centered approaches for falls-screening and prevention has promise, given numerous competing interests in clinical practice. Further research is warranted, however, specifically expanding this pilot to more clinics and better integrating information about patients who are screened including information on follow-up for outcomes. In addition, more research is needed to better understand how patients can become agents of change in their health care, including related to fall risk and prevention.

## Data Availability

The datasets during and/or analyzed during the current study available from the corresponding author on reasonable request.
